# The impact of long‐term care on quality of life

**DOI:** 10.1002/hec.3612

**Published:** 2017-11-03

**Authors:** Julien Forder, Florin Vadean, Stacey Rand, Juliette Malley

**Affiliations:** ^1^ Quality and Outcomes of Person‐centred Care Policy Research Unit (QORU), PSSRU University of Kent UK; ^2^ Quality and Outcomes of Person-centred Care Policy Research Unit (QORU), PSSRU London School of Economics UK

**Keywords:** instrumental variables estimation, long‐term care, production functions, quality of life

## Abstract

Long‐term care services are provided to help people manage the consequences of impairment, but their impact goes beyond the meeting of basic needs. Accordingly, the main aim was to explore the marginal effectiveness of care when measured in terms of people's overall care‐related quality of life (CRQoL) and assess changes in marginal effect for increasing intensity. The associated aim was to refine and apply an observational method to estimate marginal effectiveness. A “production function” approach was used with survey data, including Adult Social Care Outcomes Toolkit‐measured CRQoL, whereby we statistically modelled the expected relationship between service utilisation rates and CRQoL. This method seeks to limit endogeneity issues by controlling on observables and using instrumental variable. Using a survey of publicly funded long‐term care service users in England, we found that community‐based long‐term care significantly improved people's CRQoL but with diminishing marginal effects and effects differentiated by baseline impairment levels. There are implications for how the care system should respond to changes in global public budgets. For example, where there is unmet need, a system aimed to maximise (unadjusted) CRQoL would put more emphasis on access (more recipients) than intensity of support compared to a system operating on a needs basis.

## 1 INTRODUCTION

Long‐term care services are conventionally provided to help people manage the consequences of impairment in undertaking activities of daily living (ADLs; Fernandez, Forder, & Knapp, 2011). Recent policy in this area has increasingly embraced the ideas of personal empowerment and independence for people with impairment and disabilities (Organisation for Economic Co‐operation and Development, 2005). Services should improve people's care‐related quality of life (CRQoL) through helping them achieve ADLs (e.g., washing, dressing, and feeding) but also by providing them with better personal contact, occupation, and control and so serving the “higher order” domains (in a Maslovian sense) of quality of life (Forder & Caiels, 2011).

In line with this focus on the individual is the idea that the performance of public care systems should be assessed by measuring their effectiveness at improving service users' quality of life. The performance of local care systems in England is assessed using a national set of indicator metrics that form the adult social care outcomes framework (Department of Health, 2014). A core indicator in that framework is the CRQoL of service user, for which a standardised multiple response, self‐reported instrument, the Adult Social Care Outcome Toolkit (ASCOT) is used (Makai, Brouwer, Koopmanschap, Stolk, & Nieboer, 2014; Netten et al., 2012). Data are collected in a national survey of local authority supported social care recipients.

The main aim of this study is to explore the marginal effectiveness of services when measured in terms of the impact on recipient quality of life. The associated aim is to refine and apply an observational method to estimate marginal effectiveness.

We focus on care provided in people's own home or local community (rather than in an institutional setting). As in many other countries, the public care system is organised with a significant degree of local autonomy (Kraus et al., 2010). The amount or intensity of care provided (usually after an assessment of need)—that is, the hours per day of home care support, numbers of visits to day centres, and the size of personal budgets—will vary between areas accordingly.

A person's quality of life, including both lower and higher order aspects, derives from a range of activities and experiences, many of which could be potentially affected by care services, depending on the nature of the care tasks undertaken. Accordingly, with regard to the main aim of the paper, we have two hypotheses. The first is that improvement in the CRQoL of an individual, when measured using ASCOT, will be positively correlated with the intensity of their care support, but that we will see diminishing marginal effects. Care services will be more effective at lower order support and attend to those tasks first before affecting high‐order outcomes. Our second hypothesis is that the marginal effectiveness of additional (units of) intensity will be greater for people with higher degrees of impairment compared to people with lower degrees. The underlying argument is that people with higher impairment have a greater capacity to benefit from care services (Netten et al., 2012; Netten & Forder, 2010).

Despite the high level of public expenditure, relatively little systematic evidence exists about the effectiveness of care services, especially when measured in final outcome terms. The existing literature on the (cost‐)effectiveness of home care has tended to focus on the effects of services in preventing the need for more intensive service options (e.g., hospitalisation or institutional long‐term care; Davies, Fernandez, & Nomer, 2000; Grabowski, 2006). The evidence for these services tends to be mixed. Others are focused on “hospital at home” services rather than services focused on supporting people with long‐term conditions (Fraser, 2003). Forder, Malley, Towers, and Netten (2014) found a positive correlation between intensity and CRQoL but did not explore the scale of diminishing effects.

Marginal productivity issues have also been considered in healthcare, and many studies have found or argued that there are diminishing marginal effects (Baicker & Chandra, 2004; Cutler & McClellan, 2001; Luce, Mauskopf, Sloan, Ostermann, & Paramore, 2006; McClellan & Newhouse, 1997; Rosen, Cutler, & Vijan, 2006; Schoder & Zweifel, 2011; Stukel, Lucas, & Wennberg, 2005). Indeed, there is some suggestion that incremental healthcare intensity produces no additional benefit—so‐called flat‐of‐the‐curve medicine (Enthoven, 1980; Fuchs, 2004). Fuchs argues that the most effective interventions would get diffused and used first. Moreover, cross‐section differences in health outcomes would be primarily determined by non‐medical factors, which can be taken as a capacity for benefit argument (although we would seek to control for non‐care service factors in our analysis below).

Regarding our second aim, the use of conventional evaluation methods such as randomised control trials (RCTs) is relatively rare in this field. There are issues about research ethics for RCTs (despite the relevance of the equipoise argument) and about the external validity of RCT results, particularly considering the complex and on‐going nature of the long‐term (Hartman, Grieve, Ramsahai, & Sekhon, 2015; Raine et al., 2016). Pragmatic alternative methods for estimating effect (and cost) that do not involve control groups are used in this study. In particular, we refine and use a “production function” observational method to assess impact using survey data, as proposed in previous work (Forder et al., 2014).

As hypothesised above, other things equal, we ought to see a correlation between long‐term care service use and (care‐related) quality of life ratings in the sample, and these production relationships can be modelled statistically. In practice, both the levels of service use and quality of life are highly correlated with the individual's and other characteristics. Sample selection (endogeneity) issues are partially addressed by controlling on observable confounders. Nonetheless, because we would expect some confounders to be unobserved in our regression models, endogeneity problems are likely to remain. Accordingly, the approach uses instrumental variables (IV) to tackle selection (of treatment intensity) on unobservables (Jones & Rice, 2011). Regarding our first hypothesis, we consider different functional forms for our regression analysis to explore the expected diminishing marginal effects. There is a substantial industrial economics literature assessing returns to scale, building on the pioneering work of Cobb and Douglas (Douglas, 1976). We take a similar approach assessing the shape of a number of general functional forms as they are fitted to the data.

This paper is an output from the *Identifying the Impact of Adult Social Care* study. A full description of the sample and associated analysis is given in Forder et al. (2015), which focused on the aim of estimating the impact of community‐based social care and providing “adjusted” indicators of impact to be used by decision makers.

## 2 METHODS

### 2.1 Service user utility

The utility of a person (*i*) with care needs—denoted by *U*_*i*_—is likely to be affected by the severity of their condition and other needs‐related circumstances ( $u_{i}^{n}$), the use of informal care (*Y*_*i*_), and the amount of care services they utilise. These services can be publicly funded ( $X_{i}^{L}$) or privately paid ( $X_{i}^{P}$). Following Becker's model of altruism in the family (Becker, 1981), the service user's utility will also be affected by the utility of informal carers (*V*_*i*_) and vice versa. Finally, the person will receive utility from non‐care aspects of their lives ( $u_{i}^{m}$). Taking these elements together gives

(1) $$U_{i}=U_{i}\left(u_{i}^{n},V_{i},Y_{i},X_{i}^{P},X_{i}^{L},u_{i}^{m}\right).$$

Similarly, the utility of any carer of person *i* would be

(2) $$V_{i}=V_{i}\left(v_{i}^{n},U_{i},Y_{i},X_{i}^{P},X_{i}^{L},v_{i}^{m}\right).$$

The amount of formal services the person uses is determined by a “needs” assessment, which in England is generally undertaken by a local authority care manager who works with the service user and their family to develop a care plan. The level of publicly funded support available is affected by the eligibility policy of the assessing authority. We denote *L*_*i*_ as the policy of the local authority responsible for person *i*, where the assessment results will depend on needs, and the family's resources, denoted by *G*_*i*_. Thus, the amount of service use is given by the vector function: $X_{i}^{L}\left(L_{i},u_{i}^{n},G_{i}\right)$, which can include a number of different service components (e.g., some home care and some day care). This variable can be regarded as being exogenous.

The amount of both informal carer support and privately paid care will be determined by a “family assessment” that will account for the outcome of the public care services assessment, $X_{i}^{L}$. In particular, we can define $Y_{i}^{*}=\text{argmax}\;V_{i}\left(Y_{i}|\;G_{i},X_{i}^{L},X_{i}^{P}\right)$ and $X_{i}^{P^{*}}=\text{argmax}\;U_{i}\left(\;X_{i}^{P}|G_{i},X_{i}^{L},Y_{i}\right)$. The function $Y_{i}^{*}$ can be used in 1, after solving for *V*_*i*_, to give a partial reduced‐form function:

(3) $$U_{i}=U_{i}\left(u_{i}^{n},v_{i}^{n},G_{i},X_{i}^{P*}\left(X_{i}^{L}\left(L_{i}\right)\right),X_{i}^{L}\left(L_{i}\right),u_{i}^{m},v_{i}^{m}\right)$$

### 2.2 Empirical analysis

This utility function 3 is the basis for an empirical model of person *i*'s current CRQoL, which we denote by $u_{i}^{c}$. Because we focus on CRQoL, $u_{i}^{m}$ and $v_{i}^{m}$ are constants that we can set to zero. Taking a linear approximation of 3, we have

(4) $$u_{i}^{c}=U_{i}\left(u_{i}^{m}=0,v_{i}^{m}=0\right)=u_{i}^{e}+\theta \left(X_{i}\left(L_{i},u_{i}^{e},G\right)\right)+\epsilon_{i}^{\mathrm{uc}},$$

where $X_{i}=X_{i}^{L}+X_{i}^{P^{*}}$, that is, the total amount of formal services being used. Also, $u_{i}^{e}=\theta_{0}+\theta_{n}u_{i}^{n}+\theta_{v}v_{i}^{n}+\theta_{G}G_{i}$ is the person's CRQoL in the absence of services (and where informal care support is at its level when no formal services are used, because the function *θ* implicitly embodies changes in informal care for different (exogenous) provision of formal services *X*_*i*_). Finally, $\epsilon_{i}^{\mathrm{uc}}$ is the error term. In practice, people with care needs will be using services, and therefore, $u_{i}^{e}$ is a hypothetical level of CRQoL that could be expected if formal services were not used. For shorthand, we refer to $u_{i}^{e}$ as the “expected CRQoL.”

As a simplifying assumption, we can define a function so that the effect of service utilisation *X*_*i*_ is linear in a coefficient *β*^*u*^, that is, $\theta_{i}\left(X_{i}\left(L_{i},u_{i}^{e},G\right)\right)=\beta^{u}x_{i}\left(X_{i}\right)$ with *x*_*i*_(*X*_*i*_) = 0 for *X*_*i*_ = 0. Using an explicit functional form requires us to make an assumption about the shape of the *x*_*i*_ function. Substituting into 4 gives,

(5) $$u_{i}^{c}=u_{i}^{e}+\beta^{u}x_{i}\left(L_{i},u_{i}^{e},G_{i}\right)+\epsilon_{i}^{\mathrm{uc}}.$$

One approach would be to estimate 5 using ordinary least squares (OLS) with a range of observable needs‐related factors as proxies for $u_{i}^{n}$ and $v_{i}^{n}$. However, in practice, we only have an imperfect observation of all the needs‐related factors in $u_{i}^{n}$ and $v_{i}^{n}$, and so in $u_{i}^{e}$, leaving unobservable needs factors in the error term that are also correlated with service levels $x_{i}\left(u_{i}^{e}\right)$. This omitted variables endogeneity bias arises because the relevant unobserved characteristics of people providing the counterfactual experience (of a different level of utilisation of services) might be different to those of people using services of amount *x*_*i*_(*X*_*i*_).

Another approach to estimate effect sizes is using self‐estimation of the counterfactual (Mueller, Gaus, & Rech, 2014). Service users are asked to estimate their own counterfactual experience of a different level of service use. Practical applications of this approach have involved asking people to rate their CRQoL in the absence of services (i.e., where *X*_*i*_ = 0), which produces an estimate of $u_{i}^{e}$. Mueller et al. (2014) recognise that because individuals have to hypothesise about the counterfactual state, some “self‐estimation bias” is possible.

This approach can be combined with (OLS) regression on observables whereby the self‐estimate of $u_{i}^{e}$ is also used as a control factor. To be explicit, suppose the observable needs‐related proxies are denoted by the vector *n*_*i*_. We can define ${\tilde{u}}_{i}^{e}$ as the self‐reported indicator of expected utility: $u_{i}^{e}={\tilde{u}}_{i}^{e}+\eta_{i}^{e}={\tilde{u}}_{i}^{e}+\beta_{n}n_{i}+\eta_{i}^{\mathrm{ne}}$ where $\eta_{i}^{e}$ is the error and $\eta_{i}^{\mathrm{ne}}$ is the error without any observable need effects. Using this in 5 gives

(6) $$u_{i}^{c}={\tilde{u}}_{i}^{e}+\beta n_{i}+\beta^{u}x_{i}\left(L_{i},u_{i}^{e},G_{i}\right)+\epsilon_{i}^{\mathrm{uc}}+\eta_{i}^{\mathrm{ne}}.$$

In principle, the estimate of $u_{i}^{e}$ embodies some information about non‐observable factors, and so this method should help reduce the chance of endogeneity bias, depending on the correlation between self‐estimation bias and the level of service use. Unfortunately, the unobserved self‐estimation bias $\eta_{i}^{\mathrm{ne}}$ is not likely to be entirely independently distributed with respect to $u_{i}^{e}$. In particular, the person's self‐estimation might be affected by the scale of their prior need and the amount of services they receive. Consequently, the error component $\eta_{i}^{\mathrm{ne}}$ may still be correlated with *x*_*i*_.

A further option is to use IV estimation. The challenge is to find good instruments, that is, factors that affect the current CRQoL score ( $u_{i}^{c}$) but only through service use (*x*_*i*_), giving an IV specification of *x*_*i*_ that is not correlated with $u_{i}^{e}$.

### 2.3 Instrumentation

Our theoretical set‐up can help guide the choice of instruments. One potential instrument is the local authority's social care policy; the term *L*_*i*_ in 5. As a result of policy preferences and exogenous supply factors, we can expect that a person whose care is organised by one local authority (LA) would receive a different level of support compared with a person with the same circumstances whose care is organised by a different LA. Moreover, this difference would not be a function of the individual's characteristics, and so have no bearing on $u_{i}^{e}$.

Rather than use a simple dummy variable for LA in the estimation, we instead created a “spatially lagged” service use variable. This is calculated as the average service utilisation amount of other people (*k*) in the sample living in the same LA as the person in question: $S\left(x_{i}^{\mathrm{IV}}\right)=\left\{\;{\bar{x}}_{k\neq i\in L_{i}}=1/\left(N_{L_{i}},-,1\right)\sum_{k\neq i\in L_{i}}x_{j}|i\in L_{i}\right\}$. The level of service use by other people in the sample (*x*_*j*_) should have no direct effect on a given service user's expected CRQoL ( $u_{i}^{e}$) but is likely to be correlated with their service use (*x*_*i*_).

We therefore undertook the following IV estimation of 6:

(7) $$u_{i}^{c}=\beta_{0}^{\mathrm{IV}}+\beta_{1}^{\mathrm{IV}}n_{i}+\beta_{2}^{\mathrm{IV}}{\tilde{u}}_{i}^{e}+\beta^{\mathrm{uIV}}{\hat{x}}_{i}\left(n_{i},{\tilde{u}}_{i}^{e},S\left(x_{i}^{\mathrm{IV}}\right)\right)+e_{i}^{\mathrm{IV}},$$

where ${\hat{x}}_{i}$ is a function of *n*_*i*_, self‐reported indicator of expected utility, ${\tilde{u}}_{i}^{e}$, and the spatial lag S $\left(x_{i}^{\mathrm{IV}}\right)$ instrument.

The spatial lag instrument seeks to capture differences in policy and supply between care authorities. However, because this variable is partly defined at the LA level, there is a possibility that it might be correlated with factors that vary on a geographical basis, where these factors could have a direct effect on CRQoL. We cannot directly test the validity of the instrument, but following the literature we assess whether the instrument is balanced with respect to the needs‐related characteristics of service users (Rahman, Norton, & Grabowski, 2016). In particular, we split the sample into two groups around the median value of IV and then test whether paired (*t* test) comparisons of the covariates, *n*_*i*_ and ${\tilde{u}}_{i}^{e}$ are significantly different between the two groups. Finding no significant difference provides some reassurance as to the validity of the instrument.

In addition, we included area level controls directly in the model: (a) dummy variables for higher level geographies and (b) a spatially lagged specification of the dependent variable. We would expect omitted geographically correlated variables to be (at least partially) captured by these variables rather than left in the error.

### 2.4 Functional form

The marginal effects of changes in the intensity of provision *X*_*i*_ will depend on the shape of the service effect function $\beta^{u}{\hat{x}}_{i}\left({\hat{X}}_{i}\right)$. We experimented with a range of functional forms of 7, including monomial and polynomial functions, the latter including piecewise polynomials in the form of cubic basis splines.

The single intensity‐variable (monomial) specifications of 7 were estimated using two stage least squares in Stata 14 with a two‐step feasible generalised method of moments estimator (to allow for arbitrary heteroscedasticity).

The polynomial specifications were estimated using the predicted value of the (manually instrumented) intensity as the subject variable in the second stage. We estimated two polynomial functions models: a 3‐polynomial model with cube‐root, linear, and cubed values of service intensity as terms; and a model with (basis) cubic splines of service intensity. In both cases, the predicted value of service intensity was as estimated, by OLS, as follows:

(8) $$X_{i}^{1/3}=\alpha_{0}+\alpha_{1}n_{i}+\alpha_{2}{\tilde{u}}_{i}^{e}+\alpha_{3}S\left(x_{i}^{\mathrm{IV}}\right)+\epsilon_{i}$$

The 3‐polynomial variant of 7 was then

(9) $$u_{i}^{c}=\beta_{0}+\beta_{1}n_{i}+\beta_{2}{\tilde{u}}_{i}^{e}+\beta_{3}\hat{X_{i}^{1/3}}+\beta_{4}{\left(\hat{X_{i}^{1/3}}\right)}^{3}+\beta_{5}{\left(\hat{X_{i}^{1/3}}\right)}^{9}+e_{i}.$$

Splines are piecewise polynomial functions that can have a simple form locally but allow global flexibility. We used cubic splines that are piecewise third‐order polynomials that pass through a set of control points. In this case, 7 becomes

(10) $$u_{i}^{c}=\beta_{0}+\beta_{1}n_{i}+\beta_{2}{\tilde{u}}_{i}^{e}+\sum_{k=1}^{N}\beta_{3k}b_{k}\left({\hat{X}}_{i}\right)+e_{i},$$

where $b_{k}\left({\hat{X}}_{i}\right)$ is the *k*th basis spline and ${\hat{X}}_{i}={\left(\hat{X_{i}^{1/3}}\right)}^{3}$. This function was also estimated using OLS with predicted values of service intensity from a first‐stage estimation.

### 2.5 Hypotheses

Our first hypothesis is supported if we find $\frac{\text{∂}u_{i}^{c}}{\text{∂}X_{i}}>0$ and $\frac{{\text{∂}}^{2}u_{i}^{c}}{{\text{∂}}^{2}X_{i}}<0$ for all observed values of *X*_*i*_. Our second hypothesis is supported in general if we find that $\frac{{\text{∂}}^{2}u_{i}^{c}}{\text{∂}X_{i}\text{∂}u^{e}}<0$ for observed values of *X*_*i*_ over the range of values of $u_{i}^{e}$. However, a more pragmatic test, which we use, is whether $\frac{\text{∂}u_{i}^{c}}{\text{∂}X_{i}}\left({\bar{u}}^{e1}\right)<\frac{\text{∂}u_{i}^{c}}{\text{∂}X_{i}}\left({\bar{u}}^{e2}\right)<\frac{\text{∂}u_{i}^{c}}{\text{∂}X_{i}}\left({\bar{u}}^{e3}\right)$ for sample relevant average values of expected CRQoL, such that ${\bar{u}}^{e1}>{\bar{u}}^{e2}>{\bar{u}}^{e3}$ (e.g., where these values are means for terciles of the expected utility distribution in the sample). A practical way to estimate the relevant coefficients is to use interaction terms in 7:

(11) $$u_{i}^{c}=\beta_{0}^{\mathrm{IV}}+\beta_{1}^{\mathrm{IV}}n_{i}+\beta_{2}^{\mathrm{IV}}{\tilde{u}}_{i}^{e}+\beta^{\mathrm{uIV}1}{\hat{x}}_{i}+\beta^{\mathrm{uIV}2}{\hat{x}}_{i}u^{e}+e_{i}^{\mathrm{IV}}$$

and compare $\frac{\text{∂}u_{i}^{c}}{\text{∂}X_{i}}\left(u^{e}\right)=\beta^{\mathrm{uIV}1}\frac{\text{∂}{\hat{x}}_{i}}{\text{∂}X_{i}}+\beta^{\mathrm{uIV}2}\frac{\text{∂}{\hat{x}}_{i}}{\text{∂}X_{i}}u^{e}$ for $u^{e}={\bar{u}}^{e1},{\bar{u}}^{e2},{\bar{u}}^{e3}$.

## 3 DATA

The main data collection in the *Identifying the Impact of Adult Social Care* study was a survey of community‐based social care service users. The sample was drawn either from a non‐stratified random sample of the eligible clients in each local authority's social care database or from those respondents from the 2012/2013 Adult Social Care Survey who indicated that they would like to receive information about follow‐up research. In total, 14,021 letters were sent out by the councils (13,654) and home care providers (367). One thousand seven hundred thirty return slips were received (12.3% response rate). Of these, 1,441 indicated an interest in participating in the study and met the study inclusion criteria. Nine hundred and ninety valid interviews were in the final dataset: 546 adults with physical disability or sensory impairment, 224 adults with mental health conditions, and 220 adults with learning disability. The majority of people sampled were interviewed face‐to‐face, whereas about a quarter were interviewed by telephone. Full details of the survey design and characteristics are available (Forder et al., 2015).

The information collected included socio‐demographic characteristics, service receipt (i.e., publicly funded, self‐funded, and informal), well‐being, health status, functional ability, control and autonomy, suitability of home and local environment for mobility needs, social contact and support, and participation in social groups (Forder et al., 2015).

This analysis used an (anonymised) sample including those with physical disability or sensory impairment and mental health. We selected people aged 40 or more, giving 729 observations, of which 699 had valid information about CRQoL. There was some missing data on a number of independent variables, with a final sample of 622 cases.

Service use intensity was measured as cost‐weighted utilisation of the main community services: home care, day care, meals, social work support, and equipment and adaptations (the total of LA‐funded and privately paid care). In this regard, England‐mean (gross) unit cost data were available. The cost‐weighted utilisation variable had the usual high degree of rightward skew. To this end, we censored all cost‐weighted utilisation values of greater than £1,500 per week (more than twice the gross cost of residential care) to be £1,500. Approximately 3% of cases were censored.

The data also included a subset of cases where the actual cost of the LA‐supported care packages was available (as supplied by the respective LA). The cost‐weighted utilisation variable (for the whole sample) was rescaled by a constant to give it the same mean as the LA package cost variable (plus 10% to allow for individual top‐up) for the subset of cases where both (non‐zero) figures were available. Individuals are free to purchase additional care privately to top‐up their publicly provided package (see Forder & Fernández, 2009, for estimates).

**Figure 1:**
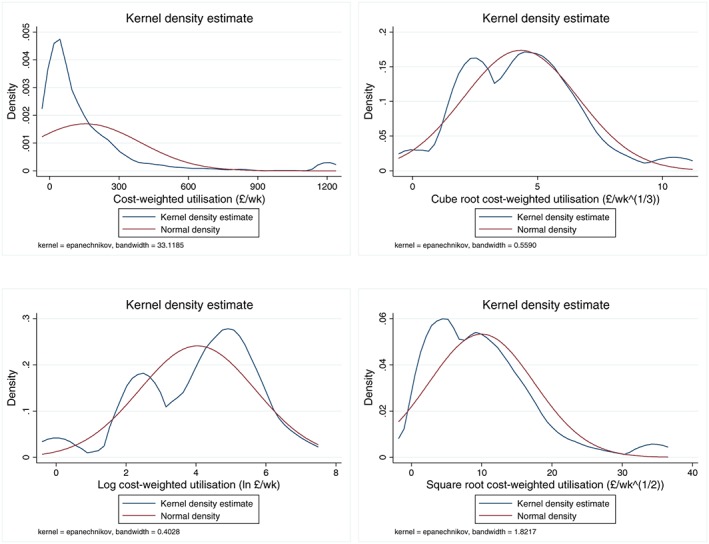
Expenditure per person (cost‐weighted utilisation)—kernel density plots

Even with censoring, cost‐weighted utilisation was highly skewed. We therefore used transformed values of *x*_*i*_ as noted above. Figure 1 shows kernel density plots. A cube root transformation appeared to best approximate the normal distribution.

**Figure 2:**
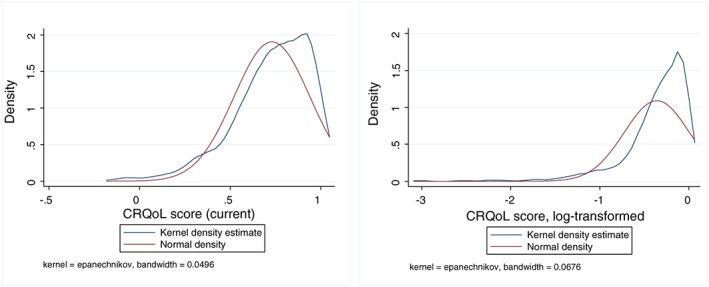
Service users' current care‐related quality of life and kernel density estimates. CRQol = care‐related quality of life

Figure 2 shows the distribution of the current CRQoL of the sampled service users, measured using ASCOT. The ASCOT indicators are preference‐weighted quality of life measures anchored around quality of life states equivalent to being dead (a score of 0) and having a score of 1 if a person chooses the highest levels on each attribute.

## 4 RESULTS

Descriptive statistics of the main variables are given in Table 1. We found the instrument to be well balanced with regard to respondent's characteristics (control variables). Table 2 has the results. Respondent characteristics were virtually identical between the upper and lower value groups of the instrument, allaying concerns that the instrument might be correlated with other, unobserved factors that directly affect CRQoL.

**Table 1:** *Identifying the Impact of Adult Social Care* study sample of service users—Descriptive statistics

Variable	Obs	Mean	*SD*	Min	Max
Endogenous variables					
CRQoL	699	0.732	0.206	−0.134	0.999
CRQoL expected	692	0.342	0.295	−0.095	0.956
CRQoL gain	691	0.391	0.278	−0.159	1.093
Cost‐weighted utilisation	691	155.554	235.754	0	1,204.464
Cost‐weighted utilisation (log)	691	4.042	1.655	0	7.095
Cost‐weighted utilisation (cube root)	691	4.331	2.297	0	10.640
Exogenous variables					
Age	717	67.838	15.077	40	90^a^
Male	729	0.422	0.494	0	1
Financial difficulties	723	0.224	0.417	0	1
Health: Good	728	0.284	0.451	0	1
Health: Fair	728	0.413	0.493	0	1
Long‐standing illness: Mental	729	0.425	0.495	0	1
ADL fail count (c7)	726	2.577	2.301	0	7
Home design: Meets needs	729	0.488	0.500	0	1
Access to local environ: Can get to places	728	0.315	0.465	0	1
Cohabiting partner	729	0.373	0.484	0	1
Education: Prof qual or degree	723	0.275	0.447	0	1
Sample: Mental Health	729	0.285	0.452	0	1
Social contact: At least weekly by phone	729	0.453	0.498	0	1
Social groups: Involved	728	0.441	0.497	0	1
Help with interview	729	0.241	0.428	0	1
CRQoL expected—easy to rate	714	0.606	0.489	0	1
Not in paid work	728	0.341	0.474	0	1
CRQoL—spatially lagged	699	0.730	0.053	0.583	0.848
Shire LA (ref met/uni LA)	729	0.535	0.499	0	1
London LA (ref met/uni LA)	729	0.141	0.349	0	1
Midlands (ref north)	729	0.246	0.431	0	1
South (ref north)	729	0.368	0.482	0	1
CRQoL expected	692	0.342	0.295	−0.095	0.956
Spatial lag cost‐weighted utilisation, PDSI, age 40+ (log)	729	5.100	0.416	3.167	5.617

*Note*. CRQoL = care‐related quality of life; ADL = activities of daily living; PDSI = physical disabilities and sensory impairments.

a Age data censored at 90+.

**Table 2:** Balance tests—*t* tests of control variables between upper and lower median groups of the instrument

	Mean		Diff	*t*	Pr(*T* < *t*)	Pr(*T* > *t*)	Pr(|*T*| > |*t*|)
	IV above median (*n* = 311)	IV below median (*n* = 311)					
Age	67.17	68.42	1.25	1.03	0.85	0.15	0.31
Male	0.43	0.41	−0.02	−0.41	0.34	0.66	0.69
Financial difficulties	0.22	0.23	0.00	0.10	0.54	0.46	0.92
Health: Good	0.27	0.28	0.01	0.36	0.64	0.36	0.72
Health: Fair	0.41	0.45	0.05	1.13	0.87	0.13	0.26
Long‐standing illness: Mental	0.43	0.43	0	0	0.5	0.5	1
ADL fail count (c7)	2.55	2.68	0.13	0.69	0.75	0.25	0.49
Home design: Meets needs	0.49	0.47	−0.02	−0.48	0.32	0.68	0.63
Access to local environ: Can get to all the places	0.29	0.32	0.03	0.79	0.78	0.22	0.43
Cohabiting partner	0.36	0.37	0.00	0.08	0.53	0.47	0.93
Education: Prof qual or degree	0.26	0.27	0.01	0.36	0.64	0.36	0.72
Sample: MH	0.28	0.28	0.00	0.09	0.54	0.46	0.93
Social contact: At least weekly by phone	0.43	0.47	0.05	1.13	0.87	0.13	0.26
Social groups: Involved	0.43	0.46	0.03	0.64	0.74	0.26	0.52
Help with interview	0.22	0.24	0.02	0.57	0.72	0.28	0.57
CRQoL expected—Easy to rate	0.61	0.61	0	0	0.5	0.5	1
Not in paid work	0.35	0.33	−0.03	−0.68	0.25	0.75	0.50
CRQoL expected	0.35	0.32	−0.02	−1.02	0.15	0.85	0.31

*Note*. CRQoL = care‐related quality of life; ADL = activities of daily living.

### 4.1 Monomial function analysis

The main model 7 results are given in Table 3, using the person's current CRQoL score as the dependent variable with cube root‐transformed utilisation as the indicator variable (Model 1). We did not use logged dependent variable specifications (including log–log [Cobb–Douglas] forms) in the main model because the CRQoL includes negative values and the logged distribution was further away from the normal distribution compared to the linear version—see Figure 2.

**Table 3:** Impact of services and support on care‐related quality of life—Cube root transformed utilisation (Model 1)

	Main Model 1		Model 1 first stage	
	Coefficient	*Z*	Coefficient	*Z*
Utilisation				
Cost‐weighted utilisation (cube root)	0.075**	2.36		
Control factors				
Age (in decades)	0.001	−0.17	−0.061	−0.99
Male	−0.016	−0.90	0.232	1.54
Financial difficulties	−0.070***	−3.31	0.170	0.86
Health: Good	0.067**	2.57	0.435**	1.98
Health: Fair	0.091***	4.49	0.175	0.94
Long‐standing illness: Mental	−0.040**	−2.18	−0.118	−0.67
ADL fail count (c7)	−0.029*	−1.94	0.454***	10.67
Home design: Meets needs	0.055**	2.47	0.472***	3.10
Access to local environ: Can get to all places	0.050***	2.67	0.121	0.65
Cohabiting partner	0.050	1.60	−0.766***	−4.58
Education: Prof qual or degree	−0.035*	−1.66	0.329*	1.90
Sample: MH	−0.035*	−1.67	−0.182	−0.96
Social contact: At least weekly by phone	0.047***	2.88	−0.109	−0.76
Social groups: Involved	0.030*	1.69	0.266*	1.83
Help with interview	−0.024	−0.98	0.467**	2.38
CRQoL expected—easy to rate	0.034*	1.90	0.287*	1.81
Not in paid work	−0.023	−1.05	0.207	1.12
CRQoL expected	0.361***	4.57	−2.315***	−7.57
Area controls				
CRQoL—Spatially lagged	−0.165	−0.75	−1.608	−0.70
Shire LA (ref met/uni LA)	−0.031	−1.40	−0.107	−0.51
London LA (ref met/uni LA)	−0.056	−1.53	−0.074	−0.23
Midlands (ref north)	0.076**	2.55	−0.384	−1.64
South (ref north)	−0.007	−0.32	0.027	0.13
Constant				
Constant	0.390*	1.75	2.017	1.06
Instrument				
Spatial lag CW utilisation, PDSI, age 40+ (log)			0.592***	3.28
				
	Statistic	Prob	Statistic	Prob
*n*	622		622	
*R* ^2^ (adjusted)	0.134		0.42	
*F* test	12.930***	<0.01	15.910***	<0.01
Weak instruments	10.788			
Endogeneity	6.613**	0.01		

*Note*. CRQoL = care‐related quality of life; ADL = activities of daily living; PDSI = physical disabilities and sensory impairments.

* 10% significance level,

** 5% significance level,

*** 1% significance level.

In line with our first hypothesis, we found a significant positive effect of service utilisation on quality of life (CRQoL). The control factors also had the expected signs. The table includes the first‐stage estimation results and shows the significance of the instrument and the strong effects of frailty (ADL count and expected CRQoL) and poor health. The instrument did not appear “weak” with an *F* test exceeding the conventional threshold of a score of 10. We also found strong support for the utilisation variable being endogenous.

Regarding area controls, we found that a number of the dummy variables for higher level geographies were significant in the second stage but not the first. The spatially lagged specification of the dependent variable was not significant at either stage.

Variant specifications were estimated. Table 4 gives results for specifications with different transformations of the utilisation variable, estimated with a generalised method of moments IV model. Although all three specifications were significant, the log form produced the least probability of information loss, measured using both the Akaike information criterion and the Bayesian information criterion. A linear specification was also estimated, but the (censored) high values of utilisation caused problems—see Figure 1. Dropping the outliers produced a better fit, but the Akaike information criterion and Bayesian information criterion were substantially higher than for the non‐linear versions.

**Table 4:** Impact of services and support on CRQoL—Alternative functional form

	Cost‐weighted utilisation		Weak instruments	Endogeneity	AIC	BIC
	Coefficient	*Z*				
Model 1: CRQoL with cube root utilisation	0.075**	2.36	10.79	6.613**	251.5	−140.7
Model 2: CRQoL with log (natural) utilisation	0.075**	2.59	19.91	6.60**	377.4	−266.6
Model 3: CRQoL with square root utilisation	0.031**	2.04	5.93	6.93***	−48.3	62.5

*Note*. CRQoL = care‐related quality of life; AIC = Akaike information criterion; BIC = Bayesian information criterion.

* 10% significance level,

** 5% significance level,

*** 1% significance level.

Table 5 gives results with different specifications of the control variables. We estimated models without the area dummies but found little difference. In another specification, we dropped the self‐reported health variable for comparison, but this made no qualitative difference to the result.

**Table 5:** Impact of services and support on CRQoL—Alternative specifications

	Cost‐weighted utilisation		Weak instruments	Endogeneity
	Coefficient	*Z*		
CRQoL with cube root utilisation without area controls	0.054**	2.06	12.72	4.04**
CRQoL with log (natural) utilisation without area controls	0.055**	2.15	21.32	4.13**
CRQoL with cube root utilisation without self‐reported health	0.062*	1.90	9.17	3.28*
CRQoL with log (natural) utilisation without self‐reported health	0.060**	2.02	19.93	3.21*
Expected CRQoL with cube root transformed utilisation	0.015^N^	0.32	8.51	1.63ᴺ

*Note*. CRQoL = care‐related quality of life.

* 10% significance level,

** 5% significance level,

*** 1% significance level.

Finally, we estimated an IV model with absent‐service CRQoL as the dependent variable. In theory as outlined above, absent‐service CRQoL should not be a causal function of the intensity of service utilisation. Nonetheless, there remains a possibility that people's judgement of absent‐service CRQoL is biased by their situation with regard to using services; for example, people with a high intensity of service use might rate their expected CRQoL to be lower (poorer) than people with more modest levels of service use. In this case, however, we found no significant effect.

Notwithstanding the issues with using a log‐transformed dependent variables, for comparison a log–log (Cobb–Douglas) version of the main model was estimated. We found a coefficient on (log) intensity of 0.15, and this supports our hypothesis of decreasing returns to scale.

### 4.2 Polynomial functional forms

The results for the cube powers polynomial and B‐splines models are given in Table 6. Regarding the cubic spline model, after some experimentation we estimated a model with 5 knots, set at the minimum and maximum values, and the three tercile points of the predicted value of cube‐root service intensity variable after re‐transforming it back to the linear.

**Table 6:** Impact of services and support on care‐related quality of life—Polynomial specifications

	B‐splines model		Cube powers polynomial	
	Coefficient	*Z*	Coefficient	*Z*
Utilisation				
Predicted cost‐weighted utilisation (cube root, re‐transformed to linear):				
Spline at £–36 (INCOMPLETE)	−0.254	−1.10		
Spline at £36	0.129*	1.85		
Spline at £83	0.217**	2.31		
Spline at £158	0.291**	2.45		
Spline at £647	0.885***	4.06		
Spline at £1,136 (INCOMPLETE)	2.011***	2.79		
Cost‐weighted utilisation—predicted (cube root) £s per week			0.082***	2.76
Cost‐weighted utilisation—predicted (linear) £000s per week			−0.242	−0.84
Cost‐weighted utilisation—predicted (cubed) £000,000s per week			0.002***	3.18
Control factors				
Age (in decades)	0.001	0.09	0.001	0.19
Male	−0.018	−1.25	−0.019	−1.38
Financial difficulties	−0.073***	−4.10	−0.073***	−4.10
Health: Good	0.059***	2.64	0.058**	2.57
Health: Fair	0.087***	5.02	0.085***	4.96
Long‐standing illness: Mental	−0.039***	−2.66	−0.039***	−2.62
ADL fail count (c7)	−0.031**	−2.47	−0.032**	−2.51
Home design: Meets needs	0.051***	2.85	0.049***	2.76
Access to local environ: Can get to all the places	0.049***	3.35	0.048***	3.31
Cohabiting partner	0.056**	2.12	0.059**	2.22
Education: Prof qual or degree	−0.038**	−2.31	−0.038**	−2.31
Sample: MH	−0.031*	−1.86	−0.032*	−1.92
Social contact: At least weekly by phone	0.050***	3.90	0.050***	4.00
Social groups: Involved	0.030**	2.12	0.029**	2.03
Help with interview	−0.023	−1.11	−0.024	−1.20
CRQoL expected—easy to rate	0.030**	2.01	0.030**	2.03
Not in paid work	−0.023	−1.28	−0.024	−1.31
CRQoL—spatially lagged	−0.195	−1.20	−0.188	−1.15
Shire LA (ref met/uni LA)	−0.032*	−1.70	−0.033*	−1.73
London LA (ref met/uni LA)	−0.059*	−1.95	−0.061**	−2.01
Midlands (ref north)	0.076***	3.13	0.077***	3.18
South (ref north)	−0.006	−0.31	−0.006	−0.34
CRQoL expected	0.366***	5.51	0.368***	5.54
Constant	0.520***	3.34	0.392**	2.33

*Note*. CRQoL = care‐related quality of life; ADL = activities of daily living.

* 10% significance level,

** 5% significance level,

*** 1% significance level.

B‐splines were generated using the *frencurv* command in Stata 14 and included one upper and one lower out‐of‐range spline. The spline based at a service intensity of zero was omitted as the base category. The specific form of 10 that we estimated was

(12) $$u_{i}^{c}=\beta_{0}+\beta_{1}n_{i}+\beta_{2}{\tilde{u}}_{i}^{e}+\beta_{31}b_{1}\left({\hat{X}}_{i}\right)+\sum_{k=3}^{7}\beta_{3k}b_{k}\left({\hat{X}}_{i}\right)+e_{i}.$$

The splines $b_{k}\left({\hat{X}}_{i}\right)$ were set at £–36, £36, £83, £158, £647, and £1,136 (with the spline at £0 excluded).

The coefficients for the B‐splines model can be interpreted as the change (gain) in CRQoL at the listed service intensity as compared to zero utilisation. Note that for these polynomial functions, the standard errors were not corrected for use of (stochastic) predicted values from the first‐stage estimation.

**Figure 3:**
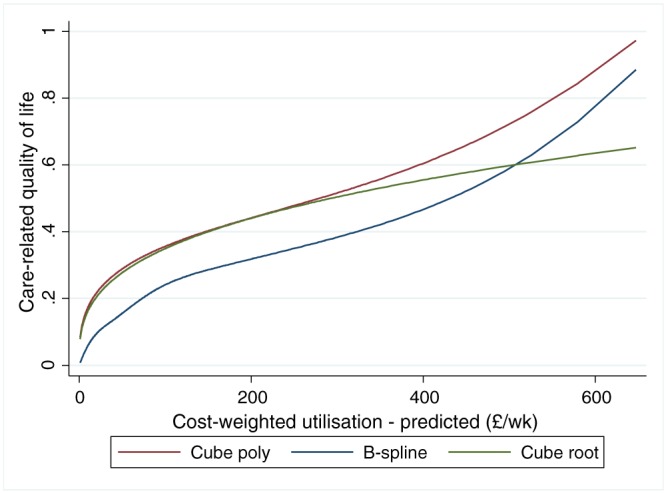
The impact of utilisation on the quality of life of cared‐for people—Alternative functional forms

Figure 3 shows these results graphically. The base cube root specification—as reported in Table 3—is also displayed for comparison. The splines version showed a very similar functional form to the cube powers polynomial, although with smaller effect sizes. All three versions showed a significant diminishing effect in the main, a result which is consistent with our first hypothesis. The more flexible forms suggest a small increasing marginal effect for changes in utilisation intensity above around £300 per week. This intensity is, however, the 90th percentile of the distribution, so the result should be treated with caution.

It is also worth noting that for service intensities above minimal levels, the size of the estimated marginal changes in CRQoL were very similar between model results with alternative functional form assumptions (as in Figure 3). More generally at service intensities of more than around £25 per person per week, marginal effects were very similar in size between models.

### 4.3 Mediating effects of impairment

As is clear from the first‐stage results as reported in Table 3, the intensity of service use is strongly positively correlated with the severity of the person's condition. Regarding our second hypothesis, after some experimentation we used an interaction term variant, 11, of the cube‐powers polynomial model, 9, by adding the terms $\beta_{w}{\left(\hat{X_{i}^{1/3}}\right)}^{n}u_{i}^{e}$ for each *n* = 1, 3, 9.

**Figure 4:**
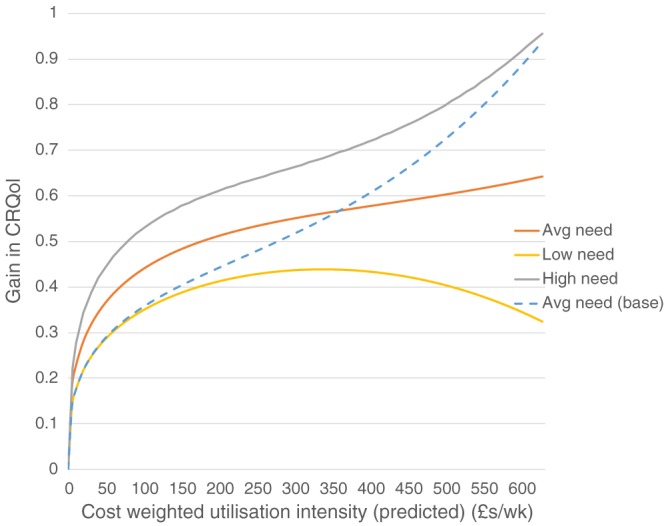
The impact of utilisation on quality of life—Interactions with need. CRQol = care‐related quality of life

The results are summarised in Table 7. An *F* test rejected the hypothesis that the interaction terms have zero effect (*F* = 2.77, *p* = .041). The results are also plotted in Figure 4, with expected CRQoL in the equations set at three values: the mean value for the whole sample (i.e., average need); the mean for people in the lower half of the distribution below the median (i.e., high need); and the mean for people in the upper half of the distribution (i.e., low need). The pattern is consistent with our second hypothesis. We should also note that low‐need people use only about half as much service intensity (an average of £90 per week) compared to high‐need people (£220 per week). In other words, low‐need people are clustered at the left‐hand side of the utilisation intensity distribution whereas high‐need people are more often further up the distribution.

**Table 7:** Impact of services and support on care‐related quality of life (CRQoL)—Interaction model

	Coefficient	*Z*
Utilisation		
Cost‐weighted utilisation—predicted (cube root) £s per week	0.143***	2.80
Cost‐weighted utilisation—predicted (linear) £000s per week	−1.009	−1.43
Cost‐weighted utilisation—predicted (cubed) £000,000s per week	0.002*	1.94
Cost‐weighted utilisation—predicted (cube root) £s per week × expected CRQoL	−0.105*	−1.84
Cost‐weighted utilisation—predicted (linear) £000s per week × expected CRQoL	1.207	1.21
Cost‐weighted utilisation—predicted (cubed) £000,000s per week × expected CRQoL	−0.005	−1.15

* 10% significance level,

** 5% significance level,

*** 1% significance level.

## 5 DISCUSSION

Regarding our first aim, we found that community‐based long‐term care in England does significantly improve the care‐related quality of life of service users (CRQoL); marginal effects are generally reduced with additional intensity of support; and people with more severe conditions (higher need) show greater marginal improvements than people with lower need, other things equal.

The second aim was to further explore the feasibility of using a production function approach and, in particular, the viability of using IV estimation to address the endogeneity or selection issues inherent in this method. We found that spatially lagged service intensity appeared to be a good instrument in this case. It was well balanced on observables and was not weak.

There are a number of general limitations of the analysis to discuss. First, although IV is commonly used, it has well‐known sensitivity to specification, particularly of the instruments. Although we have tested different functional forms, a second issue is that this method does rely on assumptions we make in this regard. Choice of functional form will particularly affect extrapolations (of service intensity) in the range with low observation density in the sample. We did not systematically compare functional forms, but the range of forms tested produced similar marginal effect estimates.

### 5.1 Resource allocation

**Figure 5:**
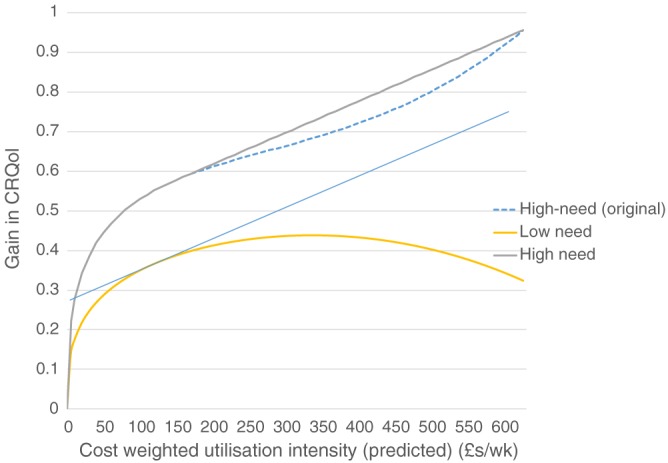
The impact of utilisation on quality of life—By need group, linear approximation for high‐need and high‐intensity service use. CRQol = care‐related quality of life

The results can be used to inform policy about prioritising public funding between different potential care recipients. Much will depend on the exact goals of the care system, but we can consider a number of scenarios where a system with a policy goal of maximising CRQoL would lead to a different allocation of resources compared to a system that allocated on the basis of need. We use the results from Figure 4 above to illustrate a number of example scenarios, with the caveat that we are using point estimates. Specifically, we use those results discounting the increasing effect after the 90th percentile, instead approximating a linear function after the turning point using the improvement value at the 95th percentile as the reference point ‐ see Figure 5.

To begin with, we can consider a scenario about the allocation of a fixed budget among a given population with care needs divided equally between high‐ and low‐need people equivalent to our sample. Given the same total budget for the sample (£10,026,640 = 310 per week per person × 622 × 52 weeks), the optimal solution (on the basis of our point estimates) gives approximately £190 per week to high‐need people and £120 per week to low‐need people. Compared to the observed allocation (£220 per week and £90 per week, respectively), this solution does shift more support to the low need group, but the change is relatively small. As such, this result does not provide a strong case for reallocating resources between given high‐need and low‐need populations.

In the above example, we worked from a given budget and a fixed number of people by need group. Another scenario is where the budget for care services is not fixed but rather set according to the opportunity costs of public expenditure more generally. Specially, suppose there is some externally determined opportunity cost threshold. With a fixed population, we would determine the optimal where the last £1 spent on care gave the same marginal benefit as the alternative; that is, $\frac{\text{∂}u_{i}^{c}}{\text{∂}B}\left(X_{i}^{*}\right)=\frac{\text{∂}u_{i}^{c}}{\text{∂}v}\frac{\text{∂}v}{\text{∂}B}=\beta^{u}x^{\prime }\left(X^{*}\right)=1/\lambda$, where the latter opportunity benefit term is conventionally expressed as the (inverse) cost of increasing CRQoL by 1 or *λ*. For example, at a *λ* threshold of £30,000 per extra CRQoL year gained, the optimal level of funding for the high‐need group would be £75 per person per week, and the low‐need group would be £50 per person per week (as based on point estimates). Seemingly, this result implies that the budget could be reduced, but it does not account for changes in access and therefore changes in the number of people served overall.

Accordingly, a third scenario is the optimal response to changes in current budget, which involves changes in access (numbers of service users) or intensity. Regarding the former, consider the value of providing the mean level of support compared to a counterfactual of no service to a new service user. By definition, this counterfactual yields zero benefit (CRQoL gain). In this case, a new high‐need service user would receive a mean level of service of £230 per week that would yield an extra CRQoL of 0.64, that is, an equivalent incremental cost‐effectiveness ratio (ICER) of slightly under £19,000 per extra CRQoL. For a change in the number in the low‐need group, the equivalent ICER is just under £15,000 per extra CRQoL (0.34 for £90 per week). This scenario would be relevant where there is unmet need, that is, a true counterfactual of no service use.

By contrast, marginal changes in service intensity around the mean (i.e., $\frac{\text{∂}u_{i}^{c}}{\text{∂}B}\left({\bar{X}}_{i}^{*}\right)$ as above) are estimated to be producing CRQoL adjusted years at more than £65,000 for high‐need groups and £45,000 for low‐need groups (using point estimates). The ICERs are quite different for intensive and extensive margins because although the mean packages are cost‐effective when compared to a zero package, they are not cost‐effective against a slightly reduced intensity. The differences in these results underlines the strong diminishing marginal effects found in the data. The problem is that we lack good information about the intra‐marginal benefits of care.

Conditional on assumptions about unmet need, nonetheless, the results suggest that a system aimed to maximise (unadjusted) CRQoL would put more emphasis on access (more recipients) than intensity of support compared to a system operating on a needs basis, although much would depend on the exact policy targets of the care system. As regards the policy implications for managing a reduced public budget, when taking a CRQoL maximising perspective, the above (ICER) results would suggest the better approach would be reductions in service intensity at the margin because they have a much lesser effect on quality of life than intra‐marginal reductions of the same amount of money. It is worth noting, however, that it may well not be easy in practice to make marginal changes to care package intensity, as care planning and assessment may introduce stickiness or lumpiness in allocating care intensity.

We have only considered (unweighted) CRQoL impacts on service users. For a more comprehensive assessment, we would also want to factor in the collateral benefits of services for informal carers and perhaps allow the possibility that the CRQoL for people with high levels of need might attracted a greater weighting than for low‐need groups.

## 6 CONCLUSION

There is a lack of evidence on the impact and cost‐effectiveness of long‐term care services for people with disability and frailty. Conventional service evaluation studies are few in number. This paper has sought to provide estimates in this regard, exploiting observational data techniques, which proved to be feasible. Within a context of tight public funding for care services and the need to maximise the benefit of those resources, these results should provide guidance for policymakers.

## ETHICAL STATEMENT

Ethical approval for the study was granted by the Social Care Research Ethics Committee on 15 March 2012.

## CONFLICT OF INTEREST

The authors have no conflict of interest.

## FUNDING INFORMATION

This work was funded by the Department of Health, Policy Research Programme. The Grant was for the Policy Research Unit in Quality and Outcomes of person‐centred care (QORU; no. PRP100/0001).
